# The diversity of lepidopteran spatial orientation strategies – neuronal mechanisms and emerging challenges in a changing world

**DOI:** 10.1007/s00359-025-01780-3

**Published:** 2025-11-07

**Authors:** Robin Grob, Max S. Farnworth, Jacqueline Degen, Eric Warrant, Stephen H. Montgomery, Basil el Jundi

**Affiliations:** 1 Department of Biology, Norwegian University of Science and Technology, Høgskoleringen 5, 7941 Trondheim, Norway; 2 School of Biological Sciences, University of Bristol, Bristol, UK; 3 Institute of Biology and Environmental Sciences, Carl Von Ossietzky Universität of Oldenburg, 26129 Oldenburg, Germany; 4 Department of Biology, Lund Vision Group, Lund University, Sölvegatan 35, 223 62 Lund, Sweden

**Keywords:** Compass orientation, Central complex, Dispersal, Mushroom bodies, Migration, Insect ecology

## Abstract

The Lepidoptera, butterflies and moths, display an astonishing diversity of spatial orientation strategies essential for survival, reproduction, and ecological success. These spatial orientation strategies range from basic taxes to light, wind, gravity, and chemical cues, to more advanced strategies such as straight-line dispersal, multigenerational migration across continents, and complex trap-lining foraging involving long-term spatial memory. These orientation behaviours are tightly integrated with the ecological roles of lepidopterans as pollinators, prey, and bioindicators, and are supported by a flexible neuronal network. Of special interest for successful orientation are higher-order integration centres like the mushroom bodies (centres for learning and memory) and the central complex (the centre for spatial orientation and locomotion). These centres support cue integration, compass orientation, memory, and directional decision-making. However, anthropogenic stressors, including habitat fragmentation, light pollution, pesticides, and electromagnetic noise, threaten both the environmental cues and the neural systems facilitating lepidopteran navigation, with potential cascading effects on biodiversity and ecosystem health. By combining insights from behavioural ecology, neurobiology, and conservation, we aim to provide a comprehensive overview of the challenges and adaptations that shape the navigational toolkit of lepidopterans, underlining their significance as animal models for studying spatial orientation in a changing world.

## Introduction—Lepidoptera navigating complex and changing environments

Orienting in space and navigating through complex and dynamic environments is fundamental for any animal, enabling them to find food, mates, and suitable habitats. Insects have evolved a diverse set of spatial orientation strategies that rival those of more famous navigators like migratory birds, fish, and mammals. Among them, the Lepidoptera, a taxon encompassing butterflies and moths, exhibit an impressive range of spatial orientation behaviours, from taxes, i.e. non-compass orientation (e.g. Brandt 1934), to sophisticated seasonal migrations, i.e. compass orientation (e.g. Chapman et al. 2015), and memory-based trap-lining foraging that includes positional information, i.e. navigation (e.g. Young and Montgomery 2023) (see Grob et al. 2021). These behaviours are tightly coupled to their ecological roles and life histories, making the Lepidoptera a compelling model group for studying the interplay between orientation, neurobiology, and environmental adaptation.

The Lepidoptera are ecologically indispensable. As pollinators, they support plant reproduction and biodiversity. As prey, they sustain food webs, and as bioindicators, they reflect ecosystem health and environmental change (e.g. New 2023). Their orientation strategies are equally diverse. Both as caterpillars and adults, lepidopterans perform orientation on different scales, ranging from very short trips to far-reaching dispersal behaviours, both passively and actively (e.g. Moore and Hanks 2004; Ronce 2007). Lepidopterans also navigate under immensely different environmental conditions, e.g. day- and night-active lepidopterans, where the availability and strength of orientation cues varies vastly. Despite the fact that light levels on a moonless night can be up to 1000 million times dimmer than light levels at midday (Warrant 1999), several moths are known to orient under these dim-light conditions.

This diversity of behaviours and ecological circumstances requires complex neuronal networks adapted to their navigational needs. Recent advances in neuroethology and insect neuroscience position lepidopterans as ideal animal models to study how animals process sensory information, maintain directional headings, form memories, and adapt to environmental niches. The large diversity in lepidopteran ecology is also reflected in their neuroanatomy (e.g. Farnworth et al. 2024). In addition to adaptations of the sensory input regions – the optic and antennal lobes – integrative brain centres also need to be adapted to adaptively combine a multitude of sensory cues that allow lepidopterans to manoeuvre through a wide range of habitats based on different orientation strategies. Moreover, integrative brain centres need to match the behavioural capacity that allows butterflies and moths to memorise specific locations, and to make robust navigational decisions based on a combination of experience and innate preferences. These integrative brain centres are the mushroom bodies (the centres for learning and memory in the insect brain) and the central complex (an integration centre for compass-based navigation and locomotion, e.g. Pfeiffer 2023). Both of these centres are crucial for successful orientation (Buehlmann et al. 2020, 2023), and their activity lays the foundation for the impressive diversity of navigational strategies seen in the Lepidoptera. However, these circuits and the first integration centres face many challenges that affect the way the insect brain interprets sensory cues, and that directly impact on the activity of neurons in the brain. In particular, human-induced stressors – such as light pollution, deforestation, or the use of pesticides – endanger the abundance of lepidopterans by, among many other factors, disrupting orientation behaviours in a variety of ways.

In this review, we provide an overview of our current knowledge concerning the spatial orientation strategies found within the Lepidoptera, from behavioural ecology to neural circuitry. We highlight the diversity of orientation mechanisms and the brain structures that underlie them. By integrating behavioural, neurobiological, and environmental perspectives, we aim to provide a comprehensive overview of the lepidopteran navigational toolkit and the challenges it faces, starting from simple orientation strategies, such as taxes, to more complex orientation and navigation behaviours such as migration and trap-lining.

## The spatial orientation repertoire of the Lepidoptera

### Taxes

**Fig. 1:**
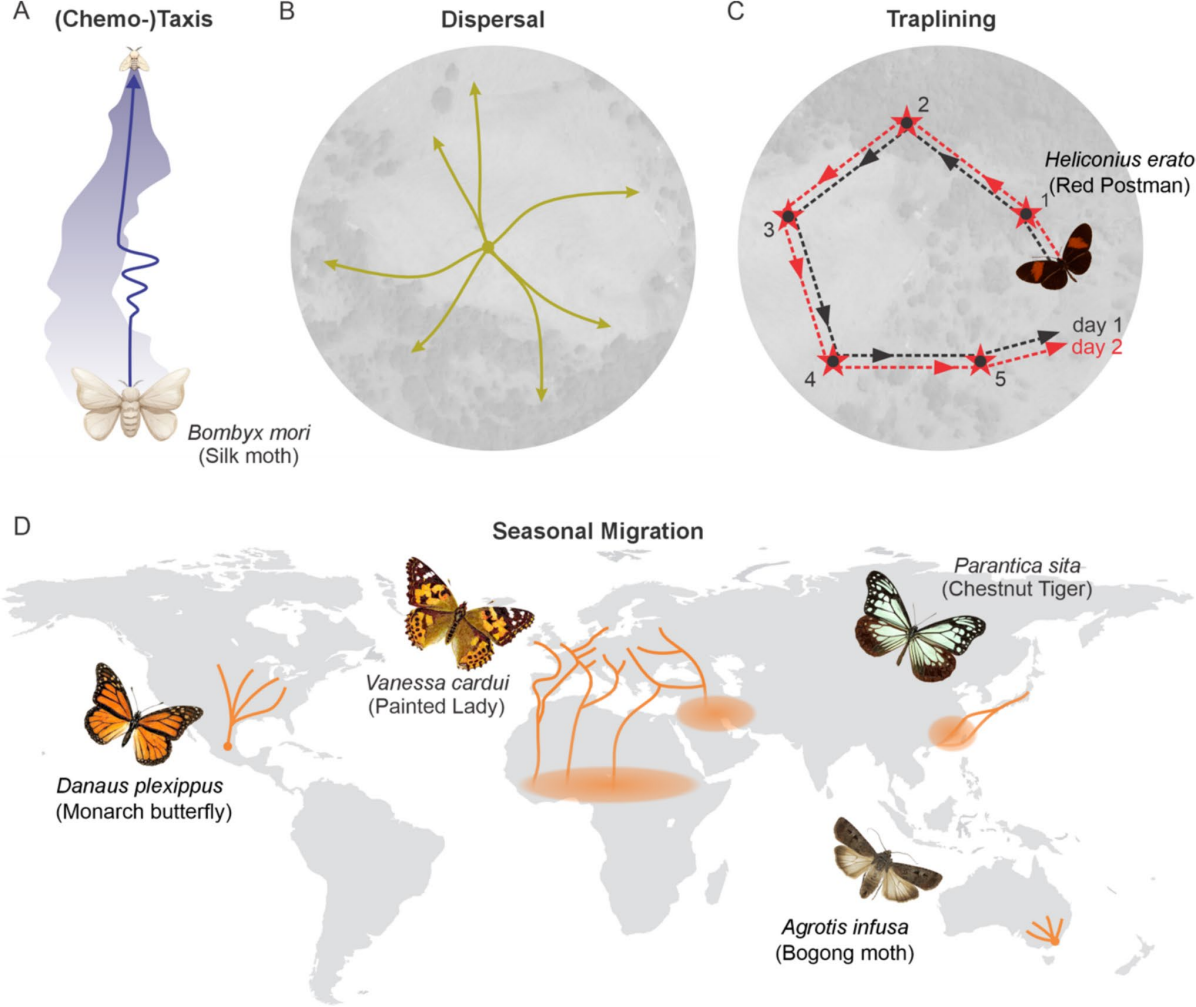
Different orientation strategies exhibited by the Lepidoptera. **A** Moths, such as *Bombyx mori*, are well known for their positive chemotaxis, as they are attracted by the pheromones released by the female specimen. If the moths lose the pheromone plume, they perform zig-zagging behaviour. **B** Butterflies are well known for their dispersal orientation strategy in which individual butterflies maintain constant flight headings into different directions, resulting in equally distributed butterflies of the same species throughout a habitat. **C** *Heliconius* butterflies are well-known for their trap-lining behaviour in which they successively visit the same locations in the same order every day. **D** Some butterflies and moths, such as the Monarch butterfly (*Danaus plexippus*) or the Bogong moth (*Agrotis infusa*) perform annual migrations to highly specific places. Many other lepidopterans, such as the Painted Lady (*Vanessa cardui*) or the Chestnut Tiger (*Parantica sita*) migrate to broad geographical regions.

“Like a moth to the flame” proverbially shows how well-known taxes are used as an orientation strategy in the Lepidoptera. Taxes are directed behaviours in response to a single environmental stimulus, either towards (positive taxes) or away (negative taxes) from the source (see Fraenkel and Gunn 1961) (Fig. 1A). This dominating cue can be of different origins, including phototaxis (in response to light), gravitaxis (also called geotaxis, in response to the gravitational force), anemo-taxis (in response to air currents), and chemotaxis (in response to chemical sources) (Grob et al. 2021). For example, even at the beginning of their lives, some lepidopteran larvae perform negative gravitaxis. By climbing in the opposite direction to the gravitational force, they avoid going to the ground and thus remain on their host plant to reach its younger leaves (Tsuji et al. 2018). As another example, moths are famously known to employ strong positive phototaxis for orientation (Brandt 1934). Positive phototaxis can be exploited in light trapping, a key tool in monitoring insect populations and in pest control. By attracting and capturing insects, light traps offer several advantages, including delaying pesticide resistance, reducing control costs, and minimizing environmental impacts (Shimoda and Honda 2013).

Additionally, moths are known for their positive chemotaxis, i.e. flying towards the source of a chemical gradient. This is often used by butterflies and moths to find host plants or mating partners, and the sex-pheromone of *Bombyx mori* was the first insect pheromone to be discovered (Butenandt et al. 1959). Male moths locate their mates using species-specific sex pheromones emitted by conspecific females (Fig. 1A). Sex pheromone signals are detected by pheromone receptors expressed in olfactory receptor neurons located in the sensillae of the male’s antennae. The signals are transmitted to the first olfactory processing centre, the antennal lobe, and are then processed further in brain regions such as the mushroom body and lateral protocerebrum (for details, see Sect. "The neuronal basis for spatial orientation in Lepidoptera") to elicit orientation behaviour towards females (Sakurai et al. 2014). Male moths are highly sensitive to the female sex pheromone. Even a single pheromone molecule can be detected by the antennae, leading to a behavioural change (Stengl 2010). The effectiveness of sex pheromone communication can be exploited as an environmentally safe management approach for pest control. This includes the use of pheromone traps, as well as pheromone-mediated mating disruption (Ioriatti et al. 2011). Wind, however, can disperse chemical signals. Male moths often perform zigzagging, i.e. oscillating flight behaviour, when following the scent trail of female pheromones to stay within the odour plume and home in on the source (Namiki and Kanzaki 2016) (Fig. 1A).To detect sex pheromones, male moths also perform a positive anemotaxis, i.e. a taxis based on air currents (Grob et al. 2021), where they fly cross wind to maximize their chances of finding the plume of a conspecific sex pheromone (Kennedy and Marsh 1974).

### Dispersal

There are also instances where simply going towards or away from a stimulus is not the right direction to choose. Due to high competition (e.g. at food sources), lepidopterans need to disperse (Fig. 1B). For efficient dispersal, animals try to leave their point of origin, for instance their larval host plant or a high density of animals, as quickly as possible (Stevens et al. 2010). The most efficient way to do so is in a straight line in any random direction. This means the animal can adopt any direction relative to an orientation cue possibly deviating from simply moving towards or away from a cue (as is the case in a taxis). Such a behaviour is called menotaxis (Grob et al. 2021). Keeping a straight line, however, is not a trivial task. While idiothetic (internal) information, i.e. proprioceptive signals from the wings and legs, can help one stay on course, it is highly prone to errors (Dacke et al. 2021). To correct these errors, idiothetic information must be integrated with allothetic (external) information. Lepidopterans use a wide range of allothetic cues for straight-line orientation, even as caterpillars (Uemura et al. 2021). These include optic flow (Baird et al. 2011; Stockl et al. 2019), celestial compass cues like the sun (Franzke et al. 2020), the stars (Dreyer et al. 2025), or the polarisation pattern in the sky (Reppert et al. 2004; Uemura et al. 2021), but also local cues like wind direction (Srygley et al. 2001), visual landmark panoramas (Franzke et al. 2020; Konnerth et al. 2023) and beaconing, also called telotaxis (Franzke et al. 2022). In this context, the cues used to keep a straight course are combined in the butterflies’ and moths’ internal compass in the brain. By comparing the current flight heading with a desired flight direction based on a short-term memory in the brain (Beetz and el Jundi 2023; Beetz et al. 2023), Monarch butterflies are able to keep their directed dispersal direction until a suitable spot is found.

Lepidopterans also use passive, often windborne, dispersal, which allows them to cover vast distances along a straight course (Clarke and Zalucki 2004; Reppert and de Roode 2018). This dispersal strategy is even used by some lepidopteran larvae (Zalucki et al. 2002). Neonate evergreen bagworms (*Thyridopteryx ephemeraeformis*) use air-borne dispersal (also called larval-dispersal, or ballooning (Bell et al. 2005)). To do so, they drop on a strand of silk and balloon on the wind. Larvae construct silken bags with fragments of plant foliage (Moore and Hanks 2004). Since the caterpillars cannot control flight direction or their goal, however, passive dispersal leads to high mortality rates (Zalucki et al. 2002).

Besides short-distance dispersal, lepidopterans also disperse on a geographical scale, driving colonisation of new locations (Ronce 2007; Stevens et al. 2010). Using this strategy, original source populations of Monarch butterflies (*Danaus plexippus*) have dispersed from North America to Central and South America as well as to Australia and the Caribbean (Clarke and Zalucki 2004; Zalucki and Clarke 2004; Merlin and Liedvogel 2019). The butterflies of these habitats are genetically separated from the North American population. In contrast to the North American population, which is famous for its annual migration, most other Monarch populations presumably consist of non-migratory butterflies (Zhan et al. 2014).

### Migration

Lepidoptera are capable of migrating over vast distances, sometimes to highly specific over-wintering or over-summering grounds (Chapman et al. 2015) (Fig. 1D). Lepidopterans, despite their small body size, can easily compete with more famous migratory animals like birds (Mouritsen 2018), when it comes to the distance they cover during their journeys. Eastern populations of the North American Monarch butterfly (*Danaus plexippus*) travel from Canada and the northern United States over 3600 km to specific overwintering grounds in central Mexico. It takes individuals about 90 days to arrive at their migratory destination (Reppert and de Roode 2018; Brower 1996). Similarly, the Australian Bogong moth (*Agrotis infusa*) migrates at night, when the availability of visual cues is diminished dramatically, flying up to 1000 km to specific aestivation caves in the Australian Alps (Warrant et al. 2016). However, the record for the furthest migratory path by a lepidopteran is held by the Painted Lady butterfly (*Vanessa cardui*). Painted Ladies are known for their trans-Saharan flights and a multigenerational cycle spanning up to 15,000 km from Afrotropical to Palearctic regions (Gorki et al. 2024) (Fig. 1D). Quite recently, it was shown that individuals found in South America migrated across the Atlantic Ocean from West Africa, with probable origins traced back to Europe. This journey covered a minimum flight distance of 4200 km over the ocean and potentially exceeded 7000 km from the point of butterfly emergence (Suchan et al. 2024), making this the longest flight of an insect ever reported.

Apart from Bogong moths and Monarch butterflies, and a small handful of other species that migrate to a highly specific destination, almost all migratory moths and butterflies journey from one broad geographic region to another. Examples for such spread-out migrations include the Painted Lady butterfly and the Chestnut Tiger butterfly (*Parantica sita*). The Chestnut Tiger butterfly uses several different migratory routes from Japan to Taiwan and mainland China (Kanazawa et al. 2015) (Fig. 1D). Lepidopteran migration most often involves a latitudinal change in one direction in spring with a reversal of this direction in autumn. To make these journeys, lepidopterans can harness favourable tailwinds that ensure efficient transport in the favoured migratory direction. Even if the wind direction and this favoured direction do not align, all but the smallest lepidopterans have the ability to fully or partially correct for the resulting crosswind drift, thereby turning and flying towards their preferred directions, greatly enhancing migratory progress (Chapman et al. 2008a, 2008b, 2010; Alerstam et al. 2011). This ability has been taken to the extreme in the huge Death’s-head hawkmoth (*Acherontia atropos*), a moth capable of migrating at night across the Alps between Europe and Africa (Menz et al. 2022). These moths can maintain a straight southward course for hundreds of kilometres irrespective of the wind direction (including when flying into headwinds). The ability of migratory lepidopterans to choose (and hold) a favoured migratory direction requires the use of one or more global compass cues (Grob et al. 2021).

Migratory butterflies and moths, just like many other insects, derive global compass information from the sky. These include the sun (Nesbit et al. 2009; Reppert and de Roode 2018) and likely the moon’s azimuthal position (Merlin et al. 2012). In addition, Monarch butterflies also possess a specific eye region, termed the dorsal rim area – like almost all insects (Labhart et al. 2009) – that analyses celestial polarised light (Stalleicken et al. 2006), but whether Monarch butterflies can use celestial polarised light for migration is still unresolved, with one study supporting the possibility (Reppert et al. 2004) and one not supporting it (Stalleicken et al. 2005). The dim polarisation pattern formed around the moon can also be used as a compass (Gál et al. 2001). Dung beetles can use the lunar polarisation pattern for short-range orientation (el Jundi et al. 2015; Foster et al. 2019; Dacke et al. 2003), but whether Bogong moths can use this pattern for long-distance navigation remains unknown. Due to the fickle nature of the moon as a celestial compass cue, it is, however, unlikely. In contrast to the moon, the stars are a much more reliable navigational cue (Foster et al. 2017, 2018; Dreyer et al. 2025).

**Fig. 2:**
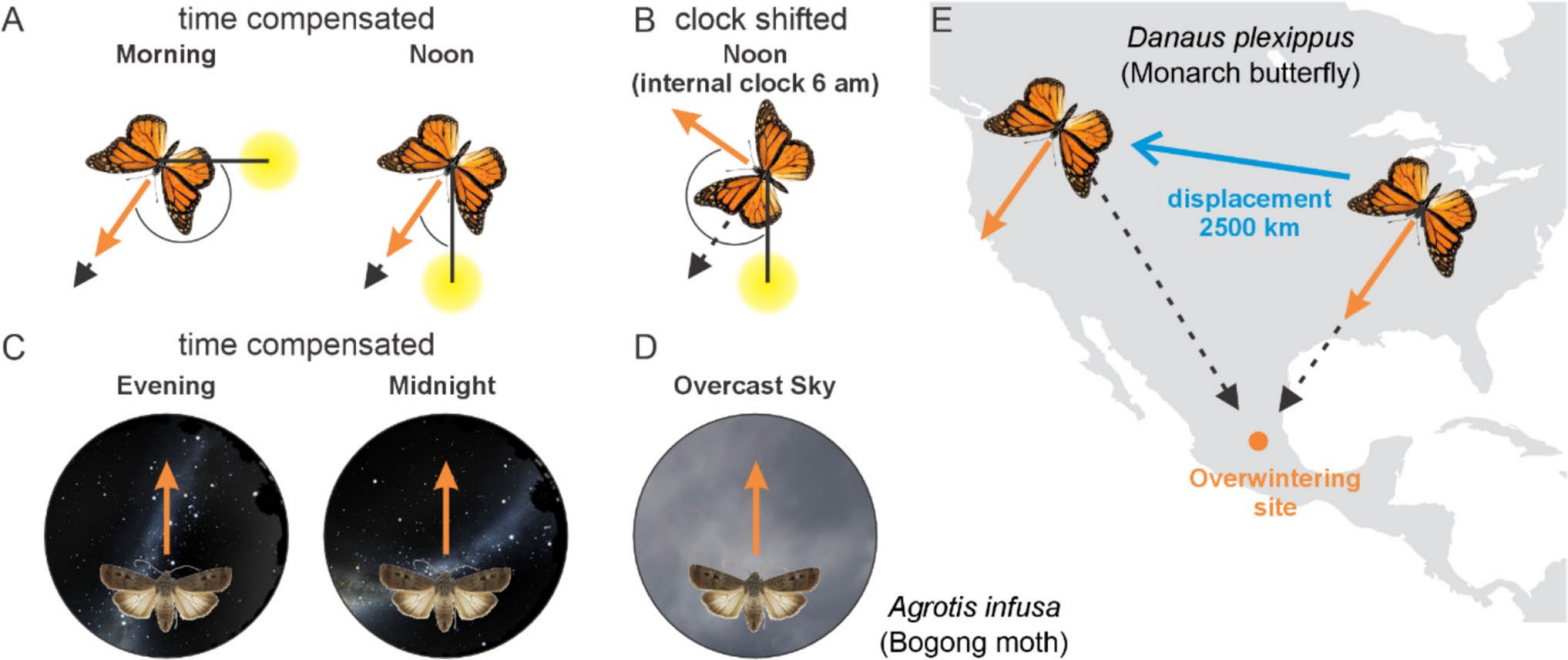
Long-distance navigational mechanisms in Monarch butterflies (**A, B, E**) and Bogong moths (**C, D**). **A** Monarch butterflies maintain the same migratory direction (south-southwest during the autumn migration) throughout the course of a day. The orange arrow indicates the current flight direction while the black dashed arrow points into the migratory direction. The solid black line indicates the direction to the solar azimuth. **B** Migratory Monarch butterflies with a clock-shifted internal clock. While the animals were tested at noon, the internal clock “predicted” that it is morning. Thus, during noon experiments the butterflies maintain the same heading relative to the solar azimuth as they would have had in the morning. **C** Stellar compass orientation in Bogong moths. Independent of the orientation of the stars (and the time of day), migratory moths maintain the same migratory direction (represented by the orange arrow) throughout the night using the stars, likely relying on a time-compensated star compass. **D** Even under a completely overcast night sky, Bogong moths are able to keep their migratory direction, suggesting that they rely on other cues, such as the Earth’s magnetic field, for migration. **E** Monarch butterflies were tested at their original location in Guelph (Ontario), where they were oriented towards their Mexican overwintering site, and then displaced and tested at a location 2500 km further west in Calgary (Alberta). After displacement, they were not able to compensate for the new location but instead kept the same heading direction they had prior to displacement. Original data A, B from Mouritsen and Frost (2002), C, D from Dreyer et al. ( 2025) and E from Mouritsen et al. (2013).

Since celestial cues change their position in a diel-, seasonal- and place-specific manner, and since migratory journeys can occur over long periods of time and across large regions of space, migratory insects need to time-compensate for these changes to use celestial cues as global cues (Grob et al. 2021; Gkanias and Webb 2025). Migratory butterflies (and hoverflies (Massy et al. 2021)) use the sun as their primary compass for long distance navigation during the day (Nesbit et al. 2009; Reppert and de Roode 2018; Warrant et al. 2016). Unlike the moon, whose position, size, brightness and presence vary extensively from day to day, the sun is a very reliable cue. At any given time of the year, it rises and sets at roughly the same places and times from one day to the next, and its trajectory across the sky varies little. Even so, as the sun traverses the sky, a flying migrant cannot simply maintain a fixed angle to the sun – this would result in a curved flight trajectory and a failure to hold a straight compass bearing towards its distant destination. To solve this problem, the migrant can use its internal biological clock to make compensatory adjustments of its flight direction according to the position of the sun at each time of the day, thus maintaining a fixed compass bearing (Fig. 2A). This has been extensively studied in Monarch butterflies (Reppert and de Roode 2018).

To hold a south-southwest flight direction during its autumn migration, eastern populations of the Monarch butterfly use a time-compensated sun compass, a fact first discovered by clock-shifting butterflies and monitoring their flight directions under natural sunny skies but at an internally “wrong time of day” (Perez et al. 1997; Mouritsen and Frost 2002) (Fig. 2B). The biological clock controlling the time compensation has been localised to the antennae (Merlin et al. 2009; Guerra et al. 2012) – butterflies whose antennae were removed, or painted black to block light input, were unable to orient to the sun in their normal south-southwest direction.

Similarly, the migratory compass of Bogong moths is unaffected by the rotation of the stars that occur as a result of the Earth’s rotation over the course of the night (Fig. 2C, (Dreyer et al. 2025)). Whether this is due to a time compensation mechanism, analogous to that found in the sun compass system of Monarch butterflies, is currently unknown.

The Earth’s magnetic field is another reliable and globally ubiquitous navigational cue for long-distance navigation, both at night and during the day. Behavioural experiments have shown that Monarch butterflies, for instance, use the geomagnetic field as a compass to fly in their inherited migratory direction, specifically using the angle (relative to the gravity vector) that field lines make with the surface of the earth (known as the inclination angle) as their directional cue (Guerra et al. 2014; Wan et al. 2021). This inclination angle allows butterflies to determine that they are flying either towards the equator (i.e. south in the northern hemisphere) or towards the pole (north) (Lohmann et al. 2022). Wan and colleagues (2021) used a behavioural paradigm, and genetic manipulations, to reveal that this inclination compass is underpinned by a violet-blue-light-dependent cryptochrome-based magnetic sense, one that is specifically mediated by the cryptochrome CRY1.

As mentioned above, Bogong moths use a stellar compass for long-distance navigation (Dreyer et al. 2025). Interestingly though, on heavily overcast nights when the stars are completely obscured, Bogong moths can still navigate in their inherited migratory direction, which suggests that they also possess a geomagnetic compass (Dreyer et al. 2025) (Fig. 2D). Preliminary evidence suggests that their magnetic sense may also be cryptochrome-based (Chen et al., in preparation). Which relevance the magnetic compass plays during nocturnal migration is still unclear, but moths may also correlate directional information derived from this compass with temporary local landmarks encountered *en route* (Dreyer et al. 2018b; Ma et al. 2025).

In addition to using the Earth’s magnetic field as a directional compass, many animals are also able to utilise the field’s local intensity and inclination angle to determine where they are within their migratory range. This “geomagnetic map” is a powerful tool in the navigational toolkit of a large variety of migratory animals (review: Lohmann et al. 2022), and animals that possess both a compass and a map are said to be “true navigators” (Grob et al. 2021). There is little evidence for the use of geomagnetic maps and true navigation in insects, but it has been revealed in another arthropod – the spiny lobster (Boles and Lohmann 2003).

Nonetheless, the possibility of true navigation has been specifically tested in Monarch butterflies using displacement experiments and investigations of historical recovery data (Mouritsen et al. 2013) (Fig. 2E). The classic experimental test of true navigation is to physically displace the navigator to a distant location, so that its normal navigational goal is now in a very different direction than it was. If the animal is a true navigator, its map sense will alert it to its new position, and the animal will turn appropriately to navigate towards its goal. If the animal is incapable of true navigation (i.e. lacks a map but has a compass), it will fail to recognise its displacement and will continue to navigate in the direction of its goal at the initial (pre-displacement) position. Mouritsen and colleagues showed that prior to displacement, Monarch butterflies were oriented in their expected south-southwest direction towards their goal in central Mexico. But when they were physically displaced by 2500 km, from eastern Canada to western Canada, they continued to fly south-southwest, even though their goal was now roughly south-southeast of them (Mouritsen et al. 2013). This result suggests that Monarch butterflies are not true navigators (but see Oberhauser et al. 2013). Once in western Canada, a true navigator would have turned and flown south-southeast (see Boles and Lohmann 2003). In addition to cues that provide information to maintain a constant migratory heading, these lepidopterans seem to rely on a yet unknown, highly specific stop signal that awaits discovery. It has been suggested that such a “stop signal” could be provided by olfactory cues e.g. from Oyamel fir trees (*Abies religiosa*) at the overwintering sites of the Monarch butterfly (Reppert, Gegear and Merlin 2010; Reppert and de Roode 2018, Guerra 2020) or by the geomagnetic field (Guerra, Parlin and Matter 2022). Indeed, preliminary work in Bogong moths has identified a specialised odour compound found in their oversummering caves which may act as an olfactory stop signal for this species. Exactly how these innately recognised cues (as well as compass directions) are inherited by naïve migrants from their ancestors is unknown. One possibility is that innately recognised cues are inherited as epigenetic memories from parents (e.g. Dias and Ressler 2014). Understanding whether this is the case in migratory moths and butterflies promises to be an exciting area for future research (Merlin and Liedvogel 2019; Warrant and Maleszka 2025).

### Trap-lining

Arguably one of the most sophisticated foraging strategies exhibited by lepidopterans is referred to as trap-lining. Trap-lining involves the memorization of spatially dispersed resources, and the establishment of a regular, repeatable foraging route between those resources (Janzen 1971; Ohashi and Thomson 2009). In contrast to the orientation strategies discussed before, trap-lining needs both directional and positional information. Therefore, it is considered navigation (see Grob et al. 2021). This strategy is most extensively studied in vertebrates (e.g. Gill 1988), and in some Hymenoptera (e.g. Janzen 1971; Saleh and Chittka 2007; Lihoreau et al. 2010) but is rarely reported in other insect taxa. Nevertheless, the foraging behaviours of multiple Lepidoptera, including males of *Jalmenus evagoras* searching for female pupae (Pierce and Elgar 1985) and *Heliconius* butterflies exploiting floral resources (Gilbert 1975), have been reported to rely on a trap-lining strategy (Fig. 1C). *Heliconius* are likely the best candidates for lepidopterans possessing similar spatial learning capacities to Hymenoptera and have been subject to the most thorough behavioural investigation (Young and Montgomery 2023).

*Heliconius* are a genus of Neotropical butterfly found across Central and South America, which underwent an adaptive radiation in the past ~ 15my, occupying a range of habitats and displaying a vast array of colour patterns (Merrill et al. 2015; Jiggins and Lamas 2016). They are also unique among butterflies, and indeed among the haustellate Lepidoptera (i.e. those lacking mandibles in the adult stage), for actively collecting and feeding on pollen grains (Young and Montgomery 2020; Gilbert 1972). Pollen provides a rich source of essential amino acids and lipids in the adult stage, when most butterflies are restricted to carbohydrate-rich, but protein-poor, nectar (Gilbert 1972). As a result, *Heliconius* have escaped the limitations of reliance on larval protein reserves for reproduction, and have evolved extended lifespans without reproductive senescence (Dunlap-Pianka et al. 1977; Foley et al. 2025), enabling them to produce many more eggs over their lifespan (Dunlap-Pianka et al. 1977; O'Brien et al. 2003; Pinheiro de Casto et al. 2025). Access to this key resource is, however, generally limited to a restricted range of floral plant species (Estrada and Jiggins 2002; Boggs et al. 1981) which are distributed in spatially complex habitats. Given the temporal stability of tropical forests, and the continuous flowering of many of their preferred pollen sources, learning the spatial location of pollen resources would likely increase foraging efficiency and individual success when foraging for competed resources (Young and Montgomery 2020). Support for this hypothesis has been provided by multiple field studies of marked butterflies, showing that an individual will repeatedly return to specific plants, at predictable times of day, over prolonged periods of time (Gilbert 1975; Ehrlich and Gilbert 1973; Murawski and Gilbert 1986; Finkbeiner 2014), and can update foraging routes when resources are no longer profitable (Ehrlich and Gilbert 1973). Foraging routes are specific to individuals and are not influenced by overlapping home ranges or shared nocturnal roosts (Ehrlich and Gilbert 1973; Murawski and Gilbert 1986; Finkbeiner 2014). Learning of resource locations by *Heliconius* is also of increased value in this genus as they are known to form stable home ranges, with strong site fidelity, meaning individuals will actively maintain a relatively fixed spatial range (Moura et al. 2022).

In bees, trap-lines are learnt, with variability in flight paths declining with experience (Kembro et al. 2019; Lihoreau et al. 2010; Saleh and Chittka 2007). Spatial memory of resources along the trapline are formed using a combination of visual landscape scenes and skylight cues (e.g. Collett et al. 2013) and are often accompanied by distinct learning or orientation flights in which exaggerated loops around the newly identified resource permit the repeated sampling of visual information (Zeil et al. 1996; Degen et al. 2016a). Through image-matching of past and present visual scenes, together with compass and skylight cues, bees can reliably return to preferred locations (e.g. Collett et al. 2013). In *Heliconius* the precise mechanisms underpinning trap-lining are not clearly understood. Nevertheless, recent experimental work in insectary cages have confirmed that *Heliconius* are indeed able to learn spatial information across a range of scales, including large, ecologically relevant distances (Moura et al. 2023; Dell’Aglio et al. 2024). They appear to do so without reliance on local landmarks, instead relying on stable landscape cues (Moura et al. 2024), and display exaggerated flight behaviours around floral resources that may be indicative of orientation flight-like behaviour (Dell’Aglio et al. 2025). *Heliconius* have also been shown to have a capacity for time-dependent associative memory (Toure et al. 2020), and are more adept at learning combinations of visual cues compared to their closest relatives (Couto et al. 2023), which may reflect an increased ability for distinguishing between similar visual cues, which can be important for image matching. Finally, *Heliconius* also show highly stable long-term memories of learnt associations but differ from their closest relatives only in the stability of visual but not olfactory memory (Young et al. 2024; Hodge et al. 2024). Combined with evidence for consistent performance in a range of other learning and memory tasks (Young et al. 2023; Moura et al. 2023; Toure et al. 2020; Hodge et al. 2024), this suggests the mechanisms that are involved in the evolution of trap-line foraging are relatively specific to visual cues, and particular learning and memory profiles (Young and Montgomery 2023).

Given reports of spatial memory in other butterflies (Konnerth et al. 2023), as well as fairly common observations of territorial behaviour (Scott 1974; Zikan 1989; Fischer and Fiedler 2001), it is likely that some degree of spatially faithful behaviour is more common across Lepidoptera, particularly longer-lived species, than currently appreciated. The precise mechanisms used are, however, unclear. *Heliconius* do, however, show many similarities in their behaviour and neuroanatomy (see below) to trap-lining Hymenoptera, suggesting the potential for convergent neural mechanisms underpinning the independent evolution of spatially faithful foraging.

## The neuronal basis for spatial orientation in Lepidoptera

**Fig. 3:**
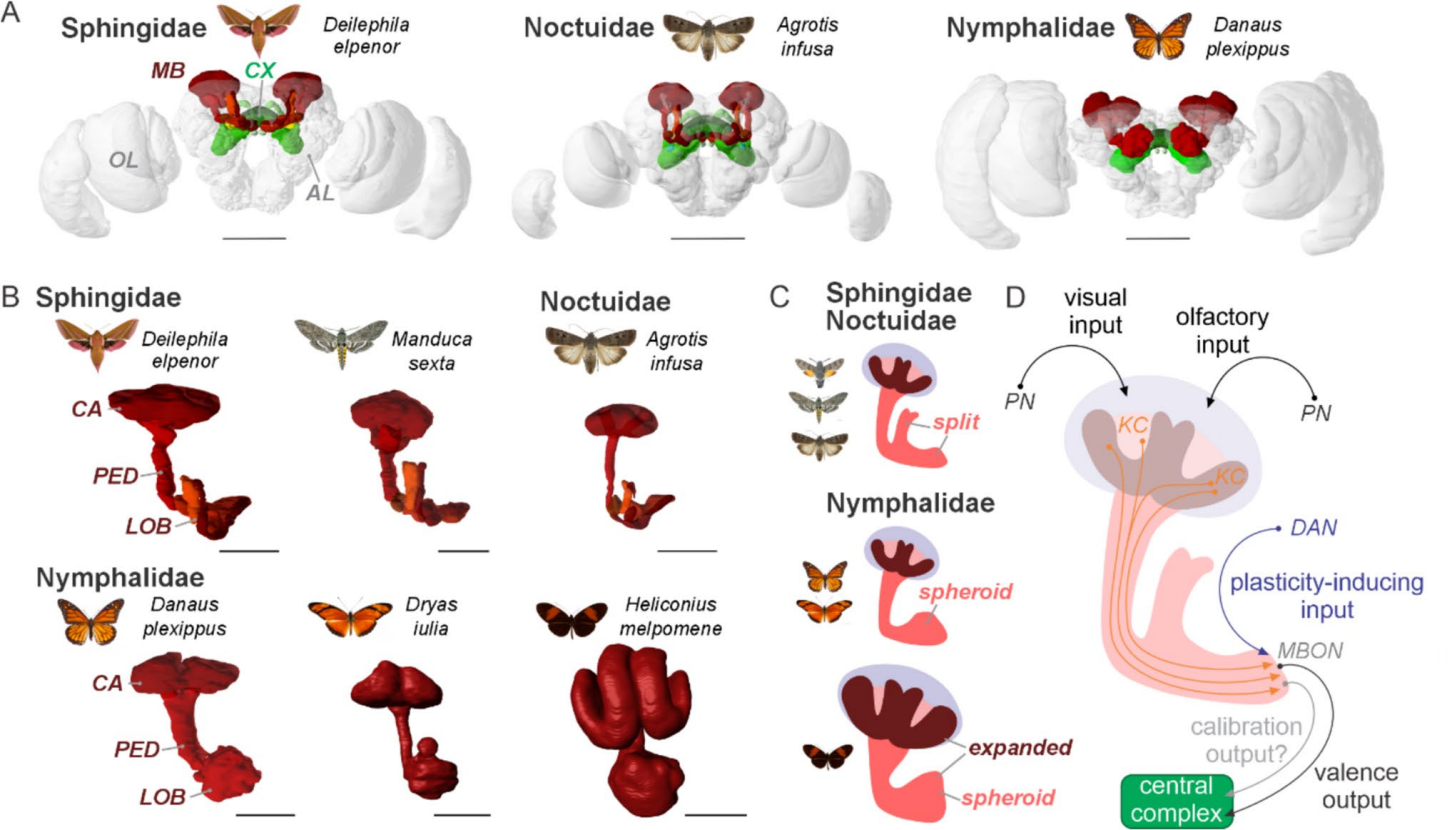
The brain of lepidopterans (**A**) and the brain region involved in learning and memory (mushroom bodies) (**B-D**). (**A**) Frontal view of three lepidopteran brains (modelled in 3D) with the mushroom bodies (MB) shown in red, and the central complexes (CX, including the lateral complexes) highlighted in green. The location of the optic lobes (OL) and the antennal lobes are indicated. Scale bars: 500 µm. (**B, C**) The diversity of MB architecture in lepidopterans. B: Dorsal view of 3D reconstruction mushroom bodies. Calyx (CA), pedunculus (PED), lobe (LOB). Scale bars: 100 µm. C: Schematic drawing of the difference in mushroom body architecture between different Lepidopteran families. **D** Schematic drawing of the neural mechanisms of the MB in insects. The MB generates a valence output signal based on multimodal inputs from projection neurons (PN). This valence signal is modified positively or negatively by dopaminergic neurons (DANs) based on e.g. experience, spatial memory, or other salient contexts. Amongst other brain regions, this valence signal is transferred via mushroom body output neurons (MBON) to the central complex, where the goal heading of an animal might be influenced. 3D models obtained from the insectbraindatabase (www.insectbraindb.org) (Heinze et al. 2021).

To orientate in space, sensory cues such as chemical and visual cues must be integrated into regions of the lepidopteran brain that allow association with past experience, circadian information, and internal representations of space (Heinze 2017). Beyond the primary processing of sensory information in more peripheral brain centres, such as in the optic lobes (OL, vision) and the antennal lobes (AL, olfaction), there are two major integrative centres in the lepidopteran brain that are associated with computational tasks required for spatial orientation and navigation: the mushroom bodies (MB) and the central complex (CX) (Fig. 3A, for abbreviations of brain regions and neurons used in this article, see Table 1).

**Table 1:** List of brain regions and neuron types (and their abbreviations) in the lepidopteran brain

Abbreviation	Explanation
AL	Antennal lobe: olfactory processing centre of the insect brain
BU	Bulb: brain area within the lateral complex
CA	Calyx: input region of the mushroom body
CX	Central Complex: brain region for spatial orientation and motor control
DAN	Dopaminergic neuron of the MB
EB	Ellipsoid body: area of the central complex (also known as CBL)
EPG	Columnar neuron: transfers signals from the EB to the PB and GA
ER	Ellipsoid body ring neuron: carries sensory signals to the EB
FSB	Fan-Shaped Body: area of the central complex (also known as CBU)
GA	Gall: brain area within the lateral complex
GPS	Global positioning system (implied in context)
KC	Kenyon cells: intrinsic neurons of the MB
LAL	Lateral accessory lobe: brain region involved in steering
LOB	Lobe: output region of the MB
LX	Lateral complex: brain region that houses the LAL, BU, and GA
MB	Mushroom body: brain centre for learning and memory
MBON	Mushroom body output neuron: transfers MB signals to other brain areas
NO	Noduli: paired neuropils of the central complex
OL	Optic lobes: visual processing centre in the insect brain
PB	Protocerebral bridge: brain area of the central complex
PED	Pedunculus: tract of the MB that houses the Kenyon cells
PFN	Columnar neuron: connects the PB with the FSB and NO
PFL	Columnar neuron: sends information from the PB and FSB to the LAL
PN	Projection neuron: carries sensory signals to the mushroom bodies
TB	Tangential neuron of the PB: CX input neuron with branches in the PB
TN	Tangential neuron of the NO: CX input neuron with branches in the NO
TU	Tangential neuron of the FSB (CBU): CX neuron with branches in the FSB

### The memory centres of butterflies and moths

The mushroom bodies are easily recognizable, enigmatic regions of the insect brain (Fig. 3A) that have been extensively studied across a number of species (Farris 2013; Strausfeld et al. 2009), including lepidopterans (e.g. Kinoshita and Stewart 2022) (Fig. 3B). Recent findings in ants and flies reveal the involvement of the mushroom bodies in spatial memory (Stern et al. 2019; Buehlmann et al. 2020; Kamhi et al. 2020; Mizunami et al. 1998). Given that lepidopterans, such as Monarch and *Heliconius* butterflies, also exhibit long-term spatial memory (Moura et al. 2023; Konnerth et al. 2023), their mushroom bodies are also highly likely to form the neural basis for memorizing visited locations based on multiple environmental cues.

To achieve this, the mushroom bodies in butterflies and moths receive multimodal – visual and olfactory – information via projection neurons (PNs, Fig. 3D). This multimodal integration occurs at the level of the calyx (CA), the input region of the mushroom bodies, which is located at the posterior end of the lepidopteran brain (Heinze and Reppert 2012; Stöckl et al. 2016; Couto et al. 2023; Kinoshita et al. 2015). There, so-called Kenyon cells (KCs) receive synaptic input and transmit information through the pedunculus (PED) to the lobes (LOB) (Fig. 3B). The output signal is then relayed to several brain regions in the lepidopteran brain, including the navigation network of the central complex (Heinze et al. 2013; Farnworth et al. 2025).

The mushroom bodies are among the most variable brain regions across the evolution of insects (Couto et al. 2023; Farris 2013; Farris and Roberts 2005; Farris and Schulmeister 2011; Farris and Strausfeld 2003; Lin and Strausfeld 2012). Their diverging relative sizes and shapes, particularly of the calyces and lobes, are indicative of underlying changes, also in Lepidoptera (Fig. 3B, C). Notably, the lobes of the mushroom bodies also show strong divergence. In Sphingidae, Noctuidae, and Bombycidae, they bifurcate into a median and a ventral lobe (Sjöholm et al. 2005; el Jundi et al. 2009; Fukushima and Kanzaki 2009) (Fig. 3B, C). In contrast, in Nymphalids and Papilionids, this obvious subdivision into distinct lobes does not exist. Instead, the pedunculus seems to terminate in a single spheroid lobe (Farnworth et al. 2024; Montgomery and Ott 2015; Heinze and Reppert 2012; Takahashi et al. 2025) (Fig. 3B, C). This shape may be linked to an increased reliance on visual input to the mushroom bodies (Couto et al. 2023; Farnworth et al. 2024). An “extreme version” of mushroom bodies in butterflies is exhibited by pollen-feeding *Heliconius*. Their mushroom bodies are massively expanded overall, reflecting a specialization in processing visual information across a greatly enlarged Kenyon-cell population (Couto et al. 2023) (Fig. 3B). This is accompanied by shifts in the proportions of KC types, particularly those that, in *Drosophila*, have been linked to visual short- and long-term memory (Farnworth et al. 2024). Thus, their memory centres may provide them with increased storage capacity, similar to what is known from hymenopterans (Ardin et al. 2016), allowing them to reliably memorize and revisit specific locations when trap-lining through cluttered forests (Fig. 1C).

### The potential neural mechanisms of spatial memory

Most insights into the neuronal mechanisms of the mushroom bodies have been developed through experiments in *Drosophila melanogaster*, aided by the precision of connectomics and the availability of tools to manipulate specific cell types within the mushroom bodies. However, through high-quality stainings and detailed anatomical assessments (Farnworth et al. 2024; Sjöholm et al. 2005; Strausfeld et al. 2009; Strausfeld 2002), we can now assume that the general logic of mushroom body circuitry is highly conserved, including in Lepidoptera.

After the Kenyon cells (KCs) receive multimodal information via projection neurons, they extract only the most pertinent sensory input. This is achieved through feedback pathways that inhibit the activity of a number of KC neurons to a given sensory modality, resulting in a sparse and highly specific coding of KC neurons to incoming sensory cues (Lin et al. 2014; Modi et al. 2020). In addition, Kenyon cells form multiple connections with different dopaminergic neurons (DANs) and mushroom-body output neurons (MBONs) in a complex array of connectivity (Heisenberg 2003; Modi et al. 2020). DANs modify the valence signals of Kenyon cells – either positively or negatively – by encoding reward or punishment signals, which are themselves based on input from the modified sensory information transduced through KCs and MBONs (Modi et al. 2020; Li et al. 2020) (Fig. 3D). MBONs, through their varied anatomical locations and projections, receive these modified valence signals from KCs and transmit them to broad regions of the brain (Li et al. 2020; Modi et al. 2020). In this way, the mushroom body network associates integrated visual and olfactory information with past experience and provides valence signals to downstream circuits. In the context of trap-lining, linking visual and olfactory cues to a specific location could allow *Heliconius* butterflies to recognize previously visited habitats and transfer information to the central complex to recall a vector that guides them to another familiar environment (Fig. 1D).

### The internal compass in the lepidopteran brain

**Fig. 4:**
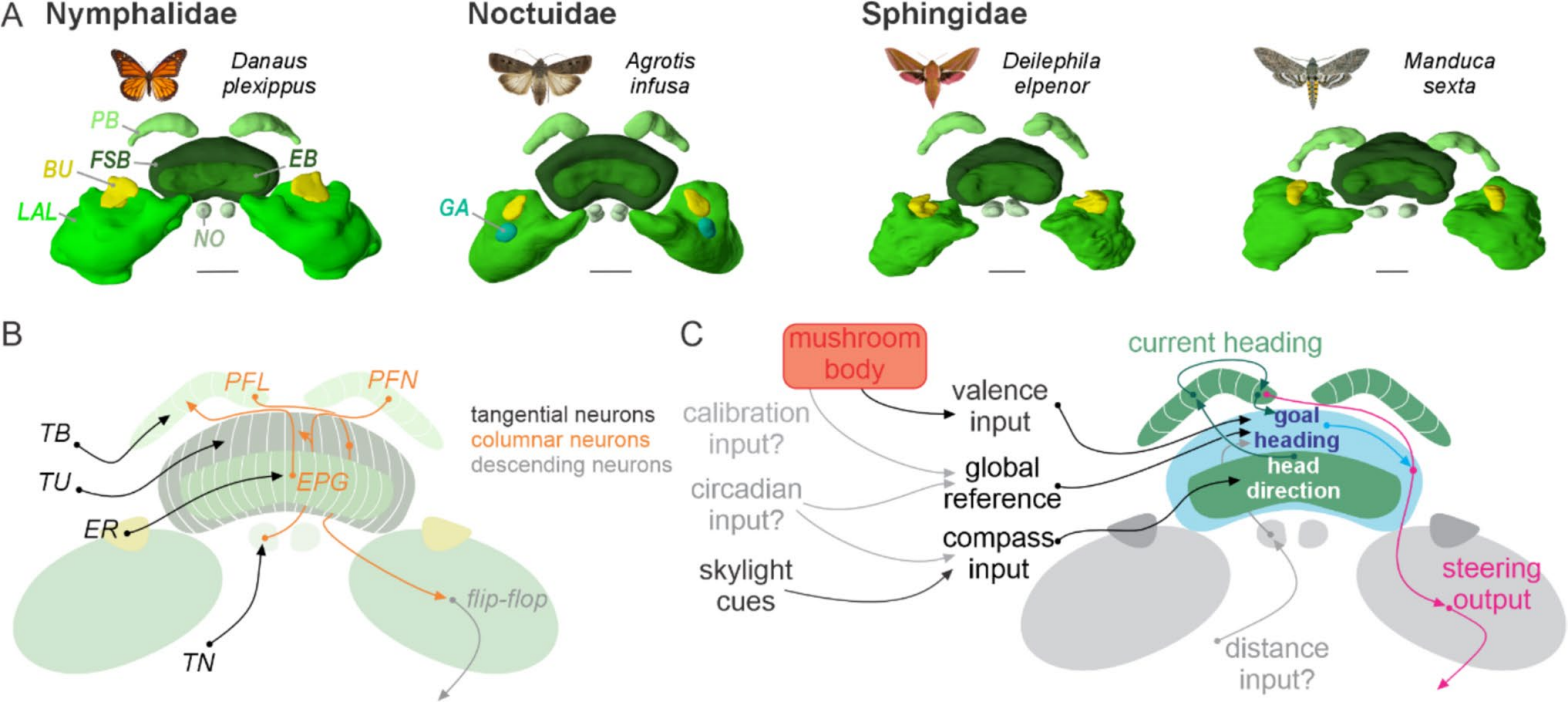
The lepidopteran central complex. **A** Fronto-dorsal view of 3D models of four central complexes, including the lateral complexes, from different lepidopteran families. The central complex is subdivided into the ellipsoid body (EB), fan-shaped body (FSB), protocerebral bridge (PB) and noduli (NO). The lateral complexes consist of the bulb (BU), the gall (GA) and the lateral accessory lobe (LAL). Scale bars: 100 µm. 3D models obtained from the insectbraindatabase (www.insectbraindb.org.) (Heinze et al. 2021). **B** Schematic drawing of the neural circuitry of the central complex. Input tangential neurons of the protocerebral bridge (TB), fan-shaped body (TU), ellipsoid body (ER), and the noduli (TN) transfer information to the central complex. EPG neurons of the ellipsoid body represent the head direction network and carry the information to the PB where the signal is transferred via PFN and PFL neurons to the fan-shaped body. PFL neurons provide synaptic output in the lateral complex where the signal is transferred to descending pathways, such as the flip-flop neurons. **C** The neural mechanisms of the central complex. Based on multimodal information, the central complex compares the current heading with a goal direction and sends steering commands to descending neurons. Information from the MB might carry valence signals to the fan-shaped body and might affect the goal heading.

The central complex has received a lot of attention over the past years due to its crucial role in controlling spatial orientation in insects, including in lepidopterans (Honkanen et al. 2019; Heinze 2024; Beetz and el Jundi 2023; Belušič and el Jundi 2024). Like in other insects, the lepidopteran central complex consists of four neuropils, the (*i*) protocerebral bridge (PB), (*ii*) fan-shaped body (FSB), (*iii*) ellipsoid body (EB), and (*iv*) the paired noduli (NO) (Adden et al. 2020; Heinze et al. 2013; de Vries et al. 2017; Farnworth et al. 2025) (Fig. 4A). Except for the noduli, the neuropils of the central complex can be divided into vertical columns in lepidopterans (Farnworth et al. 2025; de Vries et al. 2017; Heinze 2024; Heinze et al. 2013). While the neuropils of the central complex are formed through a variety of neuron types (Hulse et al. 2021; Heinze et al. 2013), their neural circuitry can be simplified to two main groups of neurons: firstly, tangential neurons, which carry signals from a number of brain regions into the central complex and, secondly, columnar neurons, which interconnect individual columns of the central complex and – at least some of them – have output branches in a closely associated region, the lateral accessory lobe (LAL) of the lateral complex (LX, Heinze et al. 2013) (Fig. 4C).

Recent neurophysiological recordings in tethered flying Monarch butterflies have provided evidence that the lepidopteran central complex is equipped with a head-direction (Beetz et al. 2022, 2023) and goal-directed steering network (Beetz et al. 2023). In addition, steering neurons transfer compensatory signals to the lateral accessory lobes, by comparing the current with the desired flight direction (Fig. 4C). In the lateral complex, this information is transferred to descending neurons that transmit the steering commands to the motor control centres in the thoracic ganglia.

Although the experiments on tethered flying butterflies do not allow for anatomical identification of the recorded neurons – due to the use of extracellular recordings – the strong physiological similarity to identified neurons in fruit flies allows us to hypothesize which columnar neurons in the lepidopteran central complex may fulfil the functions observed in these recordings. Thus, the Monarch head-direction neurons might be homologous to the *Drosophila* EPG neurons (Seelig and Jayaraman 2015; Mussells Pires et al. 2024); termed CL1a in Heinze et al. 2013), while the Monarch steering cells might be homologous to the *Drosophila* PFL3 neurons (Westeinde et al. 2024; Mussells Pires et al. 2024); termed CPU1 in Heinze et al. 2013). Taken together, the lepidopteran central complex acts like a compass, allowing butterflies and moths to constantly keep track of their heading in space, producing steering signals whenever they deviate from their flight course. Next, we will outline the sensory basis of the inputs into the central complex, how this sensory basis might vary between different lepidopterans, and how the central complex might control the wide range of orientation strategies observed in moths and butterflies.

### Multiple sensory inputs into the lepidopteran compass

Tangential neurons with synaptic output in the ellipsoid body, termed ring neurons (ER), supply the EPG compass network with sensory inputs (Fisher et al. 2019; Hulse et al. 2021). A subset of ring neurons have been extensively studied in the Monarch butterfly brain and are sensitive to celestial cues, such as the sun’s azimuth and the skylight polarisation pattern (Heinze and Reppert 2011; Nguyen et al. 2021) (Fig. 4B). In addition, these neurons are highly suited to encoding the full panoramic skyline around the animal (Nguyen et al. 2022). Thus, these input neurons provide the lepidopteran compass system with a multitude of visual cues similar to what has been discovered in fruit flies (Sun et al. 2017; Hardcastle et al. 2021). While not studied physiologically, the ring neurons have also been described anatomically in other butterflies and moths, such as in Heliconinii butterflies (Farnworth et al. 2025) and the tobacco moth (Homberg et al. 2018), indicating that the visual input pathway is highly conserved in lepidopterans, irrespective of their navigational task. In addition, recent results reported a different type of ring neuron in flies that carries wind information into the compass network (Okubo et al. 2020). If these neurons also exist in lepidopterans, they may form the ideal neural substrate to integrate visual and wind information, allowing migrating nocturnal moths to reliably derive their constant flight route despite strong winds (Menz et al. 2022).

In addition to allothetic signals, the compass also receives self-motion information, allowing the network to keep track of the animal’s heading, even in darkness (Beetz et al. 2022). Although the pathways for these idiothetic signals into the compass network have not yet been described in lepidopterans, they likely derive from proprioceptive inputs from the wings, from vestibular-like inputs from the antennae, and from motor-efference copies that predict the butterfly’s angular movements (Beetz et al. 2022). Due to this number of idiothetic inputs, the compass coding in the butterfly central complex is highly affected by the animal’s locomotory state. Thus, while in quiescence the coding of the compass is mainly dominated by allothetic inputs, the animal’s heading is based on multimodal integration of allothetic and idiothetic signals during flight, similar to what has been described in fruit flies (Seelig and Jayaraman 2015) and also for head-direction neurons in vertebrates (Taube 2007; Shinder and Taube 2014).

Although less studied physiologically, tangential neurons also provide inputs into the fan-shaped body (TU neurons), where they putatively transmit signals to networks related to (*1*) a body-invariant, global directional coding (Lu et al. 2022; Lyu et al. 2022), (*2*) networks associated with the animal’s navigational goal (Mussells Pires et al. 2024) and/or (*3*) the animal’s steering system (Westeinde et al. 2024). Remarkably, recent recordings obtained from the brain of the Bogong moth described neurons with synaptic outputs in the fan-shaped body that are tuned to the starry night sky (Dreyer et al. 2025). What role these neurons play in the navigation system of the Bogong moth is still unclear, but they might be involved in establishing a global reference system in the central complex required for long-distance migration. Taken together, the lepidopteran central complex receives a substantial amount of visual information from the animal’s environment that allows them to maintain a constant compass direction with respect to celestial and terrestrial cues. In addition to visual information, butterflies and moths are also sensitive to the Earth’s magnetic field (Dreyer et al. 2018a; Wan et al. 2021). Which (and whether) tangential input neurons encode this enigmatic cue, and where exactly magnetic information is processed in the central complex (Grob et al. 2024), is still completely unexplored.

### The potential neural basis of the orientation repertoire of lepidopterans

While the general layout of the central complex might be highly similar between different lepidopterans, other inputs, in addition to those previously mentioned, are essential for producing the different orientation strategies observed behaviourally. The neural network based on head-direction, goal-direction, and steering neurons described above is, in theory, sufficient to allow butterflies and moths to disperse in different directions based on menotaxis/ non-compass orientation (Fig. 1B). In this context, an individual-specific goal-direction tuning based on a working memory could induce different dispersal directions in different butterflies until a suitable habitat is found. Alternatively, as the EPG heading network of different individuals encodes the same environments with an angular offset, covering all directions relative to the visual scene (Seelig and Jayaraman 2015), this could inherently result in a dispersal behaviour of butterflies. In contrast, if the angular offset between the spatial tuning of the compasses of individuals is cancelled out, this could result in migrating butterflies and moths maintaining, as a population, one specific direction over longer distances (Fig. 1D). In this case, however, the directional information derived for instance from celestial cues needs to be time-compensated, allowing an animal to keep a constant travelling direction over the course of the day (Fig. 2A-C). A recent computational model suggests that this time information is integrated into the sky-compass neurons in the anterior optic tubercle, before the sun-related visual information is transferred to the lepidopteran ER neurons, and subsequently to the butterflies’ and moths’ compass (Gkanias and Webb 2025).

While there is evidence that the central complex is not essential for phototactic behaviour (Giraldo et al. 2018), recent studies show that the central complex is involved in the attraction of nocturnal moths to, for instance, odour cues. Goal-directed navigation is induced in the network of the fan-shaped body in the presence of olfactory information (Matheson et al. 2022). Thus, the lepidopteran central complex might play an important role in pheromone/odour tracking (Fig. 1A), preceding the downstream flip-flop neurons (Iwano et al. 2010) and resulting in the zigzag behaviour of moths towards a pheromone plume (Olberg 1983; Mishima and Kanzaki 1998; Namiki and Kanzaki 2016).

In addition to directional information, trap-lining as exhibited by pollen-feeding *Heliconius* butterflies (Fig. 1C) requires the central-complex network to process distance information. Interestingly, studies in fruit flies and bees have provided evidence that specific input neurons of the noduli, termed TN neurons, deliver speed/distance information to the central complex (Stone et al. 2017; Lyu et al. 2022). This information, provided by visual neurons encoding optic flow information (Milde 1993), could then be carried from the noduli to the fan-shaped body by the PFN neuron (termed CPU4 in Heinze et al. 2013) in lepidopterans, where distance information converges with the directional signals from the head-direction network (Honkanen et al. 2019). In addition, the navigation network also needs to be supplied with familiarity information about previously visited environments to allow a repeated trap-lining. Interestingly, neurons interconnecting the output region of the mushroom bodies with the fan-shaped body have been described in butterflies (Heinze et al. 2013; Farnworth et al. 2025) and might represent the neural basis for vector-based trap lining. Thus, by memorizing visited locations based on familiarity cues, such as prevailing visual information and highly specific odour cues, stored in the mushroom body network (see chapter 3.1) and transmitted as valence signals to the fan-shaped body, a vector could be recalled in the goal neurons of the central complex, allowing the pollen-feeding butterflies to navigate between different locations. Taken together, the central complex represents the navigation centre for lepidopterans which allows them to exhibit different navigational strategies based on a variety of inputs. These inputs, however, are affected by environmental circumstances to which the neuronal network has to adapt. Unfortunately, these environmental circumstances have been altered by anthropogenic influences in multiple ways, providing additional challenges to the navigational toolkit of lepidopterans.

## Emerging challenges for the navigational toolkit in a changing world

Lepidopterans are important pollinators (Balmaki et al. 2024), and their presence and diversity serve as indicators of ecosystem health. Both lepidopteran larvae and adults are vital food sources for various animals, including many species of birds, bats and mammals (Bullington et al. 2021). A decline in lepidopterans could cause detrimental changes to the natural environment and threaten global biodiversity and ecosystem health (Wagner et al. 2021; Green et al. 2021). Many species are threatened or endangered due to habitat loss, pesticide use, climate change, light pollution, and other anthropogenic stressors (Sánchez-Bayo and Wyckhuys 2019; Rumohr et al. 2023). Some of these stressors can impact the orientation success of lepidopterans directly, interfering with their dispersal, mating, host-plant selection and migration behaviour. This ultimately leads to population declines and ecological imbalances due to their harmful effects on the behaviour and physiology of individual organisms, and on interactions in communities (Diaz et al. 2019). In the following, we discuss the challenges that some anthropogenic stressors pose to the navigational toolkit of Lepidoptera.

### Light pollution

One such stressor is light pollution (Gaston et al. 2014), as it fundamentally alters the nocturnal light environment by introducing artificial illumination (Stöckl and Foster 2022; Gaston et al. 2015). Sources of artificial light include private light sources such as windows and garden lights, as well as public ones like streetlights and advertising billboards (Nachtlichter et al. 2025). In addition to such stationary sources, vehicle lights contribute to an increasing number of unpredictable, moving light sources (Bará 2025). However, the amount of light pollution at a certain location not only depends on local light sources but also on skyglow, i.e. the diffuse illumination of the night sky that impacts on the broader surroundings (Jechow et al. 2017; Cox et al. 2022). Currently, nearly half of the global land mass is affected by light pollution (Cox et al. 2022) and one can expect that artificial light emission into the environment will continue to increase (Kyba et al. 2017). Even protected areas and biodiversity hotspots cannot escape the global increase in light pollution (Guetté et al. 2018). These alterations to the nocturnal light environment disrupt the orientation cues that initiate and guide behaviour, but they also interfere with developmental and physiological processes, with wide-ranging impacts on movement, foraging, reproduction, and community ecology (Dyer et al. 2023; Owens and Lewis 2018; Bennie et al. 2016; Longcore and Rich 2004).

**Fig. 5:**
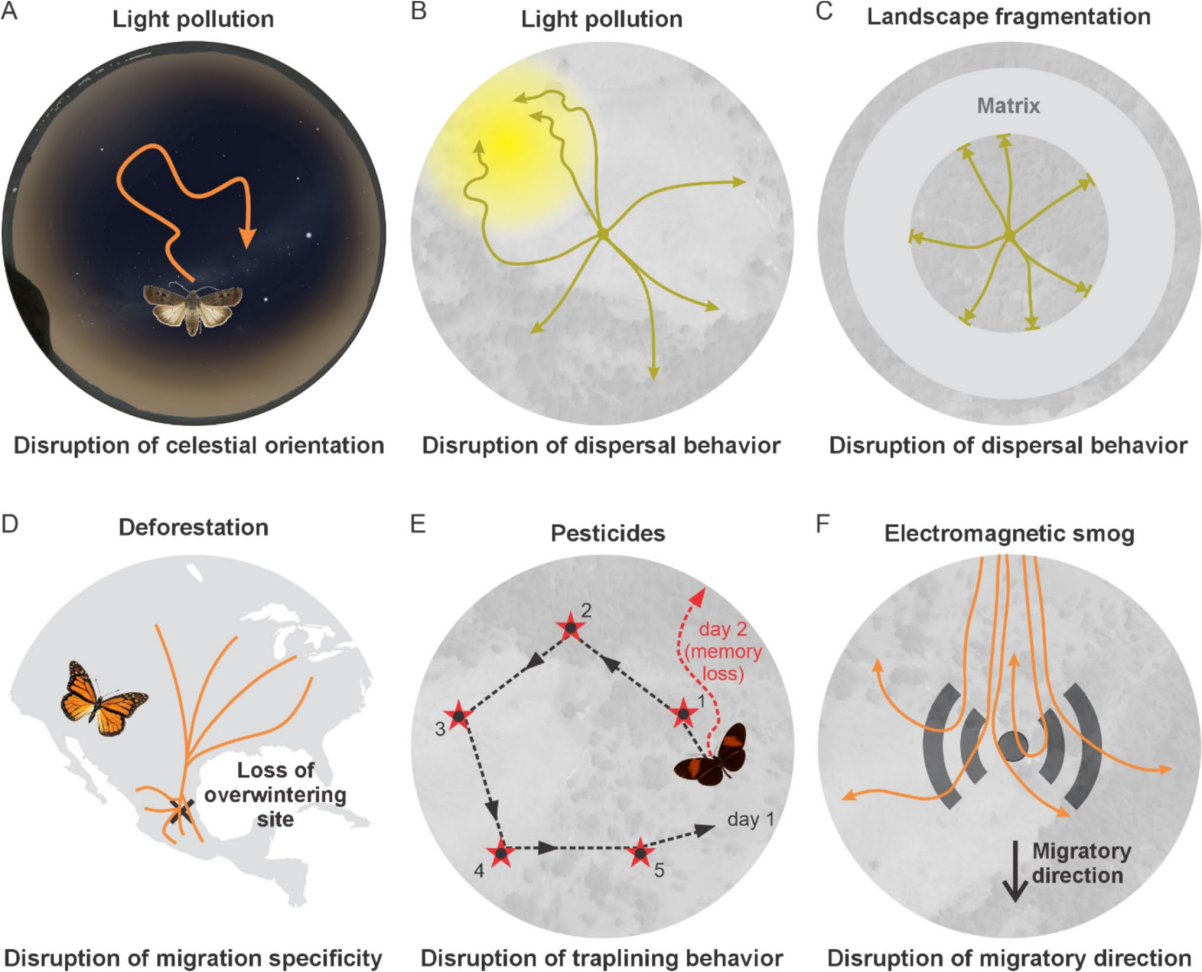
Human-made ecological challenges for the orientation compass of lepidopterans. **A, B** The orientation system of nocturnal moths is disturbed by artificial light. Skyglow can hide crucial celestial orientation cues (**A**) and local artificial light sources result in insects being trapped by the introduced light source (**B**). **C** Fragmentation of natural habitats can trap butterflies in their habitat, as they may be unable to reach new areas due to the large distances between them. **D** Deforestation of the region in Central Mexico reduces the overwintering site of the Monarch butterflies. **E** Pesticides impact the memory abilities of insects and could impact lepidopteran, such as *Heliconius*, that rely on learnt cue associations during foraging. **F** Migratory butterflies and moths relying on a magnetosensory mechanism that is disrupted by electromagnetic noise can be impacted along their migratory journey.

One way in which light pollution alters the visual environment of nocturnal arthropods is by masking important spatial cues for polarisation-guided orientation (Stöckl and Foster 2022; Kyba et al. 2011) (Fig. 5A). The moon itself, however, may be masked by artificial light near the horizon, with improved orientation performance in moths occurring only with the moon above a certain elevation threshold (Storms et al. 2022). Additionally, if exposure to artificial light at night is chronic, or occurs at sensitive times of the year, light pollution could dampen the seasonal cycles of light and dark, leading to mistimed seasonal phenology such as diapause and migration (Gaston et al. 2017; Smith et al. 2021; Agee 1969). Such a suppression of activity could trap individuals in unintended locations, delay or prevent migratory movements, and create errors in a time-corrected navigational compass. Moreover, streetlights can trap animals by inducing phototactic behaviour towards the light, instead of e.g. allowing them to disperse in a specific direction based on menotactic orientation (Fig. 1B), increase the tortuosity of moth flight paths and have a barrier effect on moths, leading to landscape fragmentation and thus hindering dispersal behaviour (Degen et al. 2024) (Fig. 5B). Among the most critical disruptions to ecosystem services is the impact of light pollution on nocturnal pollination networks (Knop et al. 2017) and diurnal plant–pollinator interactions (Giavi et al. 2021). Overall, light pollution has severe impacts across most life stages and key behaviours. These also include disruptions to reproduction, larval development, and pupal diapause, and thus might even affect day-flying insects through impacts on earlier life stages (Boyes et al. 2021).

### Deforestation and landscape fragmentation

Landscape fragmentation and deforestation are critical environmental issues. Deforestation results in the loss of approximately 10 million hectares of forest annually (FAO 2020). The consequences of deforestation are multifaceted, leading to significant biodiversity loss, disruption of ecosystems, and increased greenhouse gas emissions. In tropical forests, even selective logging, i.e. the removal of mature trees, leads to a shift in butterfly community composition, favouring generalist species over specialists (Summerville 2011). One byproduct of deforestation is landscape fragmentation. Fragmentation results in isolated habitats by presenting small lepidopterans with an impenetrable area (matrix), which can hinder species survival as well as mating success and reduce genetic diversity, by restricting dispersal behaviours (Hill et al. 2006; Storms et al. 2025; Niebuhr et al. 2015) (Fig. 5B). Remaining forest fragments may also lack the range of microhabitats necessary to support diverse communities, which can be specialised to particular sensory conditions (e.g. Wainwright et al. 2025). Moreover, predation, pollination, and nutrient cycling can be negatively affected, resulting in the disruption of critical ecosystem functions (Ditmer et al. 2021; Degen et al. 2016b; Smith et al. 2021). In addition to deforestation, agriculture, urban development, and human infrastructure expansion are primary drivers of landscape fragmentation (Burt et al. 2023).

Deforestation and landscape fragmentation have severe impacts on the navigational toolkit of lepidopterans. While Monarch butterflies show strong preference for overwintering sites with substantial forest cover, in spite of fragmentation, continued deforestation of these wintering sites could lead to the loss of overwintering-site specificity in Monarch butterflies since potential migratory “stop signals” provided by the trees might be removed (Fig. 5D). Additionally, studies have shown that moth diversity significantly decreases as forest fragmentation increases, with tree- and shrub-feeding species being particularly affected (Schmidt and Roland 2006). This decline in diversity suggests that habitat fragmentation disrupts the ecological niches that some species depend on. These effects are likely biased towards species occupying particular habitat types, such as inner forest specialists, whose habitat is degraded more rapidly during fragmentation relative to forest-edge specialists.

Whether or not populations can adapt to these changes in habitat structure is unclear. Interestingly, some studies suggest butterflies originating from fragmented agricultural landscapes exhibit superior habitat-finding abilities compared to those from continuously forested areas (Öckinger and Van Dyck 2012; Merckx and Van Dyck 2007). This enhanced ability to navigate fragmented landscapes may be an adaptive response aimed at reducing the costs associated with dispersal. Both visual and olfactory cues are crucial for habitat orientation, enabling these butterflies to effectively locate suitable habitats despite the challenges posed by fragmented environments (Öckinger and Van Dyck 2012). Relatively little work has been conducted in this area, however, and a species’ response to fragmentation may again depend on the nature of current dispersal behaviours and sensory preferences.

### Pesticides

Commonly used pesticides can have a significant impact even on non-target insect species like lepidopterans (Gilburn et al. 2015; Forister et al. 2016), including their navigational toolkit (Serrão et al. 2022). Pesticides have been shown to impact learning and memory formation in hymenopterans (Siviter et al. 2018), as well as affecting flight dynamics and reducing flight endurance (Kenna et al. 2019). Among the most widely used pesticides in the world are neonicotinoids (Bonmatin et al. 2015). They are frequently detected in soils, fresh water, and air and can have negative effects on biodiversity (Mamy et al. 2025; Forister et al. 2016; Gilburn et al. 2015). In the Large White butterfly, *Pieris brassicae*, neonicotinoids have a negative impact on pupation duration and adult body size of the butterflies (Whitehorn et al. 2018). A reduction in body size can have an impact on dispersal behaviour, with smaller butterflies being less successful in dispersing and dispersing over shorter distances (Sekar 2012; DiLeo et al. 2022; Taylor-Cox et al. 2020). The disruption of dispersal behaviour due to body size reduction can occur in combination with landscape fragmentation, further enhancing the negative effect on the navigational toolkit (Fig. 5C). Additionally, the negative impact on overall size can reduce the general capacity for long distance flight (DiLeo et al. 2022), negatively impacting the success of lepidopteran migrations.

Further, neonicotinoids have been shown to impact flight behaviours in migratory butterflies (Wilcox et al. 2021; Cibotti et al. 2024). In the Painted Lady butterfly, *Vanessa cardui*, exposure to neonicotinoids reduces free-flight metabolic rates, which has a negative influence on the flight performance especially during migration (Cibotti et al. 2024). However, critical doses and the effect on flight behaviour can be species-specific (e.g. Monarch butterflies show a high tolerance Prouty et al. 2023; Cibotti et al. 2024; Wilcox et al. 2021)) and even host-plant dependent (Prouty et al. 2021).

For honeybees it has been shown that neonicotinoids also impair memory consolidation and retrieval, posing a potential threat for foraging success (Tison et al. 2019). Neonicotinoids could disrupt memory-based foraging behaviours, as observed in species such as *Heliconius* butterflies (Fig. 5E). Neonicotinoids generally act on the central nervous system by interrupting neuronal transmission and can have lethal and sublethal impacts depending on the dose (Simon-Delso et al. 2015). This is especially problematic, since intact mushroom bodies are crucial for memory-based orientation (Buehlmann et al. 2020) (Fig. 3).

### Radiofrequency and electromagnetic noise

The magnetic sense of Monarch butterflies, and probably many lepidoptera, is based on a light-sensitive, cryptochrome-based mechanism (Wan et al. 2021). This mechanism has been shown to be disrupted in the presence of broadband radiofrequency fields in birds (Leberecht et al. 2022), but also insects (Vacha and Soukopova 2004). The magnetic sense of insects shows low sensitivity thresholds, which indicates that the almost omnipresent electromagnetic smog will have a considerable impact on the orientation capabilities of insects (Vácha et al. 2009). Radio towers for FM radio and television broadcasting send radiofrequency fields that could potentially interfere with a magnetic sense based on cryptochromes. While the role of magnetic information during the migrations of Monarch butterflies and Bogong moths is not yet fully understood, disruption of this sense could have a consequential influence on the navigational toolkit. In these lepidopterans, magnetic cues have been suggested to (1) play a role for compass orientation, (2) possibly provide positional information, and (3) possibly be involved in time-compensating the sun compass (Grob et al. 2021; Gkanias and Webb 2025) or the star compass of the nocturnal moths (Fig. 2A-C). However, the influence of radiofrequencies and electromagnetic noise on the orientation behaviour of insects has so far barely been studied (Vácha et al. 2009). Nevertheless, anthropogenic electromagnetic noise is able to disrupt magnetic compass orientation in migratory birds (Engels et al. 2014). Likewise, electromagnetic noise could prevent migratory lepidopterans from pursuing their migratory direction (Fig. 5F). Additionally, exposure to strong electric fields influences the wingbeat frequency of Cabbage Looper moths (*Trichoplusia ni*) (Perumpral et al. 1978). To better understand how electromagnetic noise might influence the navigational toolkit of lepidopterans, more research is needed to protect Lepidoptera as vital pollinators.

## Conclusion and call for action

Lepidoptera exhibit a variety of spatial orientation and navigational skills – from the precise compass-guided migrations of Monarch butterflies and Bogong moths to the intricate trap-lining foraging routes of *Heliconius*. These skills allow butterflies and moths to inhabit a wide range of environments and to effectively keep track of their heading in space during the day and at night. Given that the focus of lepidopteran research has mainly been on the orientation mechanisms related to pheromone tracking and long-distance migration, there is still a strong possibility that we have not yet grasped the full range of orientation strategies employed by lepidopterans. Moreover, it is still completely unclear how phenotypic plasticity and metamorphic development affect the navigation and orientation systems of lepidopteran brains and the external morphology of sensory systems, allowing them to extract relevant cues from their environment. Studying this in lepidopterans is especially attractive as their orientation systems rely on the use of multiple environmental cues, such as terrestrial, celestial, magnetic, and chemical cues. These sensory cues are integrated in higher-order integration centres such as the mushroom bodies and central complex, which provide lepidopterans with a navigation system based on a spatial memory centre, an internal compass, and a motor command centre. Despite the lack of work on the capacity of their memory centres, the striking visual input pathway, multimodal processing, as well as the volume of the mushroom bodies, especially in Heliconinii butterflies (Fig. 3B, C), suggest that their spatial memory abilities may rival those of hymenopterans (Young et al. 2024). Thus, lepidopterans are excellent model systems for revealing how valence signals established in the mushroom bodies are carried into the fan-shaped body, to generate robust navigational decisions within the central complex network. Recent methodological advances now permit study of these memory centres and compass circuits “in nature” and reveal how ecologically relevant conditions affect neural systems and set the challenges faced by these systems as conditions are modified by a changing environment (Cicconardi et al. 2025). This is especially important in the Lepidoptera, as they are critical pollinators (Requier et al. 2023).

A range of anthropogenic stressors such as light pollution, habitat loss and fragmentation, pesticides, and electromagnetic noise challenge the navigational toolkit of lepidopterans in multiple ways. These changes alter the environments that moths and butterflies have adapted to over millions of years and interfere with the perception and use of orientation cues. As a consequence, individuals may become disoriented, losing time and energy that could otherwise be invested in foraging or reproduction, ultimately affecting biodiversity and ecosystem stability.

To counteract these negative developments, it is essential to understand the underlying mechanisms by which anthropogenic stressors disrupt orientation. This knowledge would allow us to identify the most critical drivers of disorientation and to design targeted mitigation strategies. Importantly, stressors rarely act in isolation: urban environments often combine light pollution with noise, habitat fragmentation, and chemical exposure. Their interactive effects remain poorly understood and climate change may further exacerbate these pressures by forcing range shifts into landscapes that are increasingly fragmented and polluted. Future research therefore needs to broaden its focus to multifactorial and interactive effects, ideally under ecologically realistic conditions. Long-term monitoring and experimental field studies are crucial to assess how orientation failures scale up to affect populations and the ecosystem services they provide, such as pollination and their role as prey in trophic networks.

As lepidopterans are key pollinators, prey species, and bioindicators, their decline under anthropogenic stressors is a warning signal for broader ecosystem health (Green et al. 2021). Addressing these challenges by linking neurobiological mechanisms (such as the understanding of multimodal integration in the brain) with ecological function, research on lepidopterans not only advances our understanding of animal navigation but also helps conservation strategies aimed at safeguarding these ecologically pivotal insects in a rapidly changing world.

## Data Availability

No datasets were generated or analysed during the current study.
